# Association of the hypertriglyceridemic waist phenotype and severity of acute pancreatitis

**DOI:** 10.1186/s12944-019-1019-2

**Published:** 2019-04-09

**Authors:** Yanbing Ding, Min Zhang, Lisheng Wang, Tao Yin, Ningzhi Wang, Jian Wu, Jiehua Zhi, Weiwei Chen, Keyan Wu, Weijuan Gong, Weiming Xiao, Zhenglei Xu, Guotao Lu

**Affiliations:** 1 Department of Gastroenterology, Affiliated Hospital of Yangzhou University, Yangzhou University, No. 386 Hanjiang Media Road, Yangzhou, 225000 Jiangsu China; 2 Laboratory of Gastroenterology, Affiliated Hospital of Yangzhou University, Yangzhou University, No. 386 Hanjiang Media Road, Yangzhou, 225000 Jiangsu China; 3 Department of Gastroenterology, The second Clinical Medical College (Shenzhen People’s Hospital), Jinan University, Shenzhen, 518000 Guangdong China; 4 Department of Gastroenterology, Clinical Medical College, Yangzhou University, Yangzhou, China; 5 Department of Immunology, School of Medicine, Yangzhou University, Yangzhou, China

**Keywords:** Acute pancreatitis, Hypertriglyceridemic waist phenotype, Waist circumference index, Retrospective study

## Abstract

**Background:**

The aim of this study was to evaluate the effect of a simple visceral obesity phenotype, known as the hypertriglyceridemic waist phenotype and its quantitative indicator waist circumference index on the severity of acute pancreatitis.

**Materials and methods:**

Diagnosis and severity analysis of acute pancreatitis were determined according to the Atlanta classification guidelines, revised in 2012. We considered the hypertriglyceridemic waist phenotype as characterized by increased waist circumference and elevated triglyceride concentrations. We investigated the association between the acute pancreatitis severity and hypertriglyceridemic waist phenotype, including waist circumference index.

**Results:**

The hypertriglyceridemic waist phenotype was significantly associated with systemic inflammatory response syndrome, organ failure, and severe acute pancreatitis. The median waist circumference index and demonstration of hypertriglyceridemic waist phenotype were positively correlated with acute pancreatitis severity. In addition, multivariate logistic analysis showed that patients with the hypertriglyceridemic waist phenotype had 1.664 times the risk of organ failure and 1.891 times the risk of systemic inflammatory response syndrome, compared with the other groups.

**Conclusion:**

Upon admission, the hypertriglyceridemic waist phenotype was strongly associated with acute pancreatitis in patients. This phenotype, including waist circumference index, might be a simple method for evaluating individuals at high risk of severe acute pancreatitis.

**Electronic supplementary material:**

The online version of this article (10.1186/s12944-019-1019-2) contains supplementary material, which is available to authorized users.

## Introduction

Acute pancreatitis (AP) is an inflammatory disease of the pancreas and one of the main causes of acute abdominal pain [1]. The incidence of AP is increasing annually and ranges from 4.9 to 80 cases per 100,000 individuals worldwide [2]. AP, especially severe acute pancreatitis (SAP), imposes a serious burden on patients and society. SAP accounts for 10 to 20% of all AP cases and is often accompanied by systemic inflammatory response syndrome (SIRS) and multiple organ dysfunction syndrome, with a mortality rate of up to 30% [3, 4]. Early and accurate assessment of AP severity and prognosis is therefore of great significance for clinical diagnosis and treatment.

Recent studies have shown that abnormal lipid metabolism plays an important role in the inflammatory response and prognosis of AP and is related to conditions like obesity and hypertriglyceridemia. Obesity is a state of excessive fat tissue and is a well-known risk factor for AP that can exacerbate inflammation [5–7]. A meta-analysis showed that obesity is associated with local complications, organ failure, and high mortality in patients with AP [8].Further studies found that visceral fat, not peripheral fat, is closely related to the formation of pseudocysts in patients with AP and SIRS. In contrast, patients with excessive visceral fat are more likely to have SAP [9]. Apart from obesity; hypertriglyceridemia is another key lipid metabolism disease that affects AP [10, 11]. It has been well recognized that hypertriglyceridemia is not only the cause of AP but also a factor associated with worsened prognosis in patients with AP [12]. Clinical studies have suggested that elevated serum triglycerides (TG) are independently associated with organ failure in patients with AP [13].

The concept of the hypertriglyceridemic waist (HTGW) phenotype was put forward by Lemieux et al., who suggested that this simple phenotype can serve as a useful marker of metabolic abnormalities [14]. The waist circumference (WC) index (WTI) is a quantitative indicator of the HTGW phenotype [15]. Many previous studies have suggested that the HTGW phenotype can be used to identify the characteristics of excessive visceral adipose tissue, ectopic fat, and metabolic syndrome (Mets) [16–18]. Other studies demonstrated that the HTGW phenotype is a risk factor for cardiovascular disease and is closely related to prodromal diabetes and hypertension [19–21]. To the best of our knowledge, there is no research on the association between the HTGW phenotype and AP. The purpose of this study was to evaluate the effects of the HTGW phenotype on disease severity in patients with AP.

## Methods

### Inclusion and exclusion criteria

We retrospectively evaluated patients diagnosed with AP who were admitted to the Affiliated Hospital of Yangzhou University from January 1, 2013, to December 31, 2016. The diagnosis of AP required at least two of the following features: abdominal pain, biochemical evidence of pancreatitis (amylase or lipase elevated > 3 times above the upper limit of normal), and/or radiographic evidence of pancreatitis on cross-sectional imaging [22]. The cause of AP was considered to be biliary if gallstones or biliary sludge were observed on imaging examinations, including computed tomography (CT), magnetic resonance cholangiopancreatography, and ultrasonography [23, 24]. Hypertriglyceridemic acute pancreatitis (HTG-AP) was characterized by the presence of serum hypertriglyceridemia (≥1000 mg/dL) or by visible lactescent blood with serum hypertriglyceridemia 500–1000 mg/dL without any other causes [25–27]. We considered patients as exhibiting alcoholic AP if they consumed > 50 g per day for > 5 years, or if the patient consumed excessive alcohol shortly before the onset of AP and other causes were ruled out [28–30]. The exclusion criteria were age > 70 or < 18 years, recurrent pancreatitis, malignant tumor, ascites, pregnancy, and incomplete information. Because of the retrospective nature of this study, informed consent was waived, and the study was approved by the Ethics Committee of our hospital.

### Clinical measurements

Once admitted, each patient’s height was measured while standing with normally aligned shoulders and without shoes. The measurement was read to 0.1 cm. Body mass index (BMI) was calculated as the weight (kg) divided by height (m/m^2^) squared. Abdominal CT images were used to estimate WC. We selected the navel level on abdominal CT, and the abdominal cavity was considered an ellipse. Then the horizontal axis and vertical axis of the navel level were measured. At last, the WC (defined as the perimeter) was measured using the standard ellipse formula, as shown in Additional file 1 :Figure S1.

### Definitions of HTGW phenotype

Subjects were categorized among four phenotypes based on pre-determined cutoff points [14]. The normal waist normal TG (NWNT) group exhibited WC < 90 cm for men and < 80 cm for women, and serum TG concentrations < 1.7 mmol/L. The normal waist hypertriglyceridemia (NWET) group exhibited WC < 90 cm for men, < 80 cm for women, and serum TG concentrations ≥1.7 mmol/L). The enlarged WC normal TG (EWNT) group exhibited WC ≥90 cm for men, ≥80 cm for women, and serum TG concentrations < 1.7 mmol/L. Finally, the HTGW group exhibited WC ≥90 cm for men and ≥ 80 cm for women, and serum TG concentrations ≥1.7 mmol/L. The WTI formula was as follows: WTI (cm*mmol/L) = WC (cm) × TG (mmol/L) [15].

### The severity of acute pancreatitis

AP was categorized as mild (MAP), moderately severe (MSAP), and SAP, according to the 2012 revised Atlanta classification [28]. MSAP was associated with transient organ failure and/or local or systemic complications within 48 h. Patients with SAP exhibited persistent organ failure, involving one or more organs and lasting > 48 h.

### Systemic inflammatory response syndrome

SIRS was defined as the existence of two or more of the following [28]: (1) temperature > 38 °C or < 36 °C; (2) heart rate > 90 beats/min or hypotension (systolic blood pressure < 90 mmHg or > 40 mmHg lower than baseline); (3) shortness of breath (> 20/min) or hyperventilation (PaCO_2_ < 32 mmHg); and (4) peripheral blood leukocyte count > 12 × 10^9^/L, or neutral rod-shaped granulocyte ratio > 10%. However, other factors that could potentially cause these changes were systematically excluded.

### Organ failure

To determine organ failure, we evaluated the respiratory, circulatory, and renal systems. Organ failure was determined if the score was ≥2 points according to the revised Atlanta Classification Recommendation Marshall score system. [28]

### Statistical analyses

Statistical analyses were performed using SPSS. Descriptive statistics were used to analyze the baseline characteristics of the study population. We performed between-group comparisons of the characteristics and variables among the various severity groups. Normally distributed, continuous variables were expressed as mean ± standard deviation, and between-group comparisons were performed using an independent sample t-test; one-way ANOVA was performed when three groups were compared.

Non-normally distributed variables were expressed as medians (interquartile range) and compared using the non-parametric rank sum test. We expressed categorical data as numbers and scales, and the Kruskal–Wallis test or chi-square test was used for between-group comparisons. Categorical data were compared using the Mann–Whitney U test or chi-square test. The following indicators were selected: age, gender, BMI, hypertension, diabetes, coronary heart disease, smoking, and alcohol consumption. The HTGW phenotype was the independent variable, whereas organ failure was the dependent variable during multivariable regression analyses.

## Results

### Cohort baseline characteristics

**Fig. 1:**
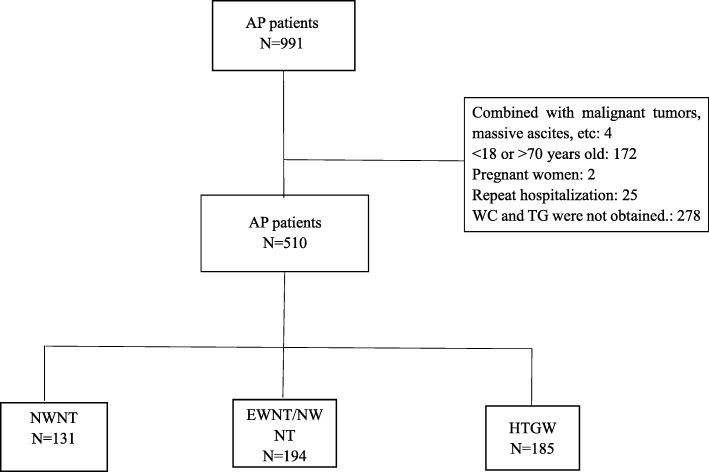
The distribution of hypertriglyceridemic waist phenotype

From January 2013 to December 2016, 991 patients were diagnosed with AP. Of these, 510 satisfied the inclusion criteria, as shown in the flowchart (Fig. 1). The patients’ demographic and clinical characteristics are shown in Table 1. The mean patient age was 47.7 ± 12.0 years, and 62.6% were male. Smokers accounted for 25.1, and 16.9% of the population reportedly consumed alcohol. Other conditions reported by patients were hypertension (7.5%), diabetes (15.9%), and coronary heart disease (2.4%).

**Table 1:** The cohort baseline characteristics

Viables	Cohort *N* = 510	NWNT *n* = 131	EWNT/NWET *n* = 194	HTGW *n* = 185	*P* value^a^
Age, years (mean ± SD)	47.9 ± 11.9	53.7 ± 11.7^#^	49.7 ± 11.4^&^	42.5 ± 10.2	< 0.001
Male (men %)	319 (62.6%)	84 (64.1%)	121 (62.4%)	114 (61.6%)	0.901
BMI*	25.5 ± 5.1	22.2 ± 5.4^#^	25.3 ± 4.0^&^	27.9 ± 4.6	< 0.001
WC	87.0 ± 9.4	78.3 ± 6.2^#^	85.6 ± 7.3^&^	94.5 ± 7.2	< 0.001
WTI (IQR)	221.7(10.7–3712.6)	68.9 (17.8–145.2)^#^	194.9(10.7–3320.0)^&^	713.7(150.8–3712.6)	< 0.001
Smoking	128 (25.1%)	29 (23.6%)	56 (31.3%)	43 (24.9%)	0.246
Alcoholic	86 (16.9%)	18 (14.6%)	36 (20.7%)	32 (18.4%)	0.412
Hypertension	38 (7.5%)	13 (9.9%)	15 (7.7%)	10 (5.4%)	0.316
Diabetes	81 (15.9%)	6 (4.6%)^#^	30 (15.5%)^&^	45 (24.3%)	< 0.001
Coronary heart disease	12 (2.4%)	4 (3.1%)	7 (3.6%)	1 (0.5%)	0.073
Etiology^#, &^					0.002
Biliary	144 (28.2%)	67 (51.1%)	58 (29.9%)	19 (10.3%)	
Alcohol	34 (6.7%)	9 (6.9%)	16 (8.2%)	9 (4.9%)	
Hypertriglyceridemia	205 (40.2%)	5 (3.8%)	76 (39.2%)	124 (67%)	
Others	127 (24.9%)	50 (38.2%)	44 (22.7%)	33 (17.8%)	

^a^represents comparing among the three groups;

^#^The HTGW compared with the NWNT group, *P* < 0.05

^&^The HTGW compared with the EWNT/NWET group, *P* < 0.05

Abbreviations: *WC* Waist circumference,*WTI* Waist circumference index,*BMI* Body Mass Index,

*IQR* interquartile range

Patients were assigned to one of three groups according to their WC and TG data. There were 131 patients in the NWNT group, 194 in the EWNT/NWET groups, and 185 in the HTGW group. The mean age for the groups was 53.7 ± 11.7 years (NWNT), 49.7 ± 11.4 years (EWNT/NWET), and 42.5 ± 10.2 years (HTGW). As expected, BMI, TG, WC, and WTI were all significantly higher in the HTGW group than in the NWNT and EWNT/NWET groups. There were no significant differences in alcohol consumption and smoking behaviors among the three groups. In terms of underlying disease, significantly more patients in the HTGW group exhibited diabetes, as shown in Table 1.

The causes of AP were biliary in 144 (28.2%), alcoholic in 34 (6.7%), hypertriglyceridemic in 205 (40.2%), and unknown in 127 (24.9%). As shown in Table 1, biliary issues were the leading cause of AP (51.1%) in the NWNT group. In contrast, the leading cause of AP in the HTGW group was hypertriglyceridemia, accounting for 67.0% and conforming to the definition of the HTGW phenotype.

### Comparison of the severity among the NWNT, EWNT/NWET, and HTGW subgroups

According to the 2012 revised Atlanta Classification system, there were 356 (69.8%) patients with MAP disease, 120 (23.5%) with MSAP disease, and 34 (6.7%) with SAP disease. The proportion of MAP decreased across the NWNT (77.1%), EWNT/NWET (73.2%), and HTGW subgroups (61.1%), whereas the proportion of SAP gradually increased from 3.8 to 10.8%. There were significant differences in AP severity among the three groups (*P* = 0.002).

As evidenced by Ranson score, the CTSI score, and the prevalence of SIRS and organ failure, patients with HTGW had more severe disease, as shown in Table 2. Furthermore, in the HTGW group, white blood cells (WBCs), and glucose were higher than the respective values in the other groups, whereas creatinine (Cr), blood urea nitrogen (BUN), and lactate dehydrogenase (LDH) did not significantly differ among the groups (Table 2).

**Table 2:** Comparison of the severity of the three groups

Viables	Cohort *N* = 510	NWNT *n* = 131	EWNT/NWET *n* = 194	HTGW *n* = 185	P value ^a^
Severity of AP^#,&^					0.002
MAP	356 (69.8%)	101 (77.1%)	142 (73.2%)	113 (61.1%)	
MSAP	120 (23.5%)	25 (19.1%)	43 (22.2%)	52 (28.1%)	
SAP	34 (6.7%)	5 (3.8%)	9 (4.6%)	20 (10.8%)	
SIRS	320 (62.7%)	32 (24.4%)^#^	63 (32.5%)^&^	95 (51.4%)	0.000
Scores of AP					
Ranson Score	1.2 ± 1.2	1.1 ± 1.1^#^	1.1 ± 1.1^&^	1.4 ± 1.2	0.032
CTSI Score	3.3 ± 2.0	2.8 ± 1.5^#^	3.3 ± 1.9	3.6 ± 2.2	0.047
Organ failure	154 (30.2%)	30 (22.9%)^#^	52 (26.8%)^&^	72 (38.9%)	0.004
Clinical indicators					
WBC (10^9^/L)	11.9 ± 4.6	10.5 ± 4.2^#^	11.8 ± 4.7^&^	12.9 ± 4.5	0.000
GLU (mmol/L)	8.8 ± 4.0	7.3 ± 2.5^#^	8.2 ± 3.3^&^	10.7 ± 4.8	0.000
Cr (umol/L)	60.8(1.5–680)	63.4(6.3–680) ^#^	60.8(1.5–430.0)	57.9(17.0–338.0)	0.017
BUN (mmol/L)	4.8 ± 2.3	5.3 ± 2.7	4.6 ± 2.1	4.6 ± 2.0	0.162
LDH (IU/L)	195.7(4.4–1461.0)	200 (4.4–786.0)	200.5(18–860)	192.0(22.8–1461.0)	0.893
TC (mmol/L)	4.4(1.1–12.7)	3.4(1.7–6.5)^#^	4.4(1.6–12.7)^&^	5.6 (1.1–12.7)	< 0.001
TG (mmol/L)	2.5(0.1–39.2)	0.9(0.2–1.7)^#^	2.4(0.1- 39.2)^&^	7.4(1.7–39.0)	< 0.001

All the shorthand is shown below: *WBC* white blood cells,*GLU* glucose,*Cr* creatinine,*BUN* blood urea nitrogen,*LDH* lactate dehydrogenase,*TC* total cholesterol,*TG* triglyceride

^a^represents comparing among the three groups;

^#^The HTGW compared with the NWNT group, *P* < 0.05

^&^The HTGW compared with the EWNT/NWET group, *P* < 0.05

### Correlation of the severity of AP with the percentage of HTGW and WTI

We compared clinical features, including the percentage of HTGW and WTI, across the MAP, MSAP, and SAP subgroups. As shown in Table 3, there were no differences in age, gender, and smoking and drinking behaviors across those with MAP, MSAP, and SAP disease. Upon examination of biochemical indicators, which helped indicate AP prognosis (WBC, LDH, Cr, and BUN), those with SAP had showed significantly higher values than the other two groups, indicating a poorer prognosis. Additionally, our study showed that there were no significant differences in BMI among the groups. However, WC and TG levels were higher in the SAP group than in the other two groups. The proportion of the HTGW phenotype grew progressively larger across the MAP (31.7%), MSAP (43.3%), and SAP groups (58.8%). We investigated WTI to further confirm the relationship between the HTGW phenotype and AP severity. Consistent with the results for the HTGW phenotype, in the SAP group, WTI was significantly higher than that in the MAP and MSAP groups.

**Table 3:** Correlation between disease severity and HTGW%、WTI

	Cohort N = 510	*n* = 356	MSAP *n* = 120	SAP *n* = 34	*P* values ^a^
Age years (mean ± SD)	47.9 ± 11.9	47.7 ± 11.8	48.1 ± 12.2	48.7 ± 11.8	0.870
Male(%)	319 (62.5%)	225 (63.2%)	75 (62.5%)	19 (55.9%)	0.701
Smoking	128 (25.1%)	82 (23.0%)	38 (31.7%)	8 (23.5%)	0.267
Drinking	87 (17.1%)	60 (16.9%)	24 (20.0%)	3 (8.8%)	0.185
BMI*	25.5 ± 5.1	25.3 ± 5.0	25.8 ± 5.6	26.4 ± 3.8	0.449
Waist circumference	87.0 ± 9.4	85.9 ± 9.1^#^	89.1 ± 10.2	90.6 ± 8.1^$^	0.000
WTI	221.7(10.7–3712.6)	209.5(15.1–3712.6)^#^	229.5(20.2-3507.0)^&^	522.8(10.7-2804.0)^$^	< 0.001
HTGW%	185 (36.3%)	113 (31.7%)^#^	52 (43.3%)	20 (58.8%)	0.001
Etiology					0.075
Biliary	144 (28.2%)	108 (30.3%)	28 (23.3%)	8 (23.5%)	
Alcohol	34 (6.7%)	26 (7.3%)	7 (5.8%)	1 (2.9%)	
Hypertriglyceridemia	205 (40.2%)	136 (38.2%)	48 (40.0%)	21 (61.8%)	
Others	127 (24.9%)	86 (24.2%)	37 (30.8%)	4 (11.4%)	
WBC (10^9^/L)	11.9 ± 4.6	11.1 ± 4.1^#^	13.6 ± 5.1	14.1 ± 5.2^$^	0.000
LDH (IU/L)	195.7(4.4–1461.0)	187.5(4.4–1461.0)	222.0(110.0–786.0)	313.0(139.0–1410.0)	0.893
GLU (mmol/L)	8.8 ± 4.0	8.3 ± 3.5^#^	9.7 ± 4.3^&^	11.6 ± 5.8^$^	0.000
BUN (mmol/L)	4.8 ± 2.3	4.5 ± 1.9	5.2 ± 3.1	5.8 ± 2.0^$^	0.039
Cr (mmol/L)	60.8(1.5–680)	60.1(1.5–128.0)^#^	63.3(17.0-181.0)	69.5(30.0–680.0)	0.017
TG (mmol/L)	2.5(0.1–39.2)	2.4(0.2–39.0)	2.5(0.2–39.2)^&^	6.0(0.1-29.4)^$^	< 0.001
TC (mmol/L)	4.4(1.1–17.1)	4.4(1.1–17.1)	4.4(2.1–12.2)	4.5(1.7–10.9)	0.679

All the shorthand are shown below: *WBC* white blood cells,*LDH* lactate dehydrogenase,*GLU* glucose,*BUN* blood urea nitrogen,*Cr* creatinine,and *TG* triglyceride

^a^represents comparing among the three groups;

^#^represents the MAP compared with the MSAP group, *P* < 0.05

^&^represents the MSAP compared with the SAP group, *P* < 0.05

^$^represents the MAP compared with the SAP group, *P* < 0.05

### Logistic regression analysis of organ failure in patients with AP

Finally, we investigated whether organ failure correlated with the epidemiology and clinical features. A multivariate logistic regression analysis was performed, and the results showed that patients with the HTGW phenotype had 1.664 times the risk of organ failure (OR = 1.664, 95% CI 1.033–2.680, *P* = 0.036) and 1.891 times the risk of SIRS (OR = 1.891, 95% CI 1.192–2.998, *P* = 0.007) compared with the other groups, as shown in Table 4. It is worth noting that patients with diabetes appear more prone to organ failure (OR = 1.722, 95% CI 1.018–2.915, *P* = 0.043) and SIRS (OR = 1.848, 95% CI 1.083–3.153, *P* = 0.024).

**Table 4:** Logistic regression analysis of organ failure and SIRS in patients with AP

Organ Failure					SIRS			
	B (S.E)	*P*	OR	95%CI	B (S.E)	*P*	OR	95%CI
Male	0.204	0.412	1.227	0.753–1.998	0.250	0.299	1.283	0.801–2.056
Age	0.014	0.157	1.014	0.995–1.035	0.000	0.965	1.000	0.981–1.020
BMI	0.019	0.406	1.019	0.975–1.066	0.051	0.035	1.052	1.004–1.103
Hypertension	−0.383	0.411	0.682	0.273–1.701	0.775	0.065	2.170	0.953–4.942
Diabetes	0.544	0.043	1.722	1.018–2.915	0.614	0.024	1.848	1.083–3.153
CHD	−0.034	0.961	0.966	0.242 ± 3.860	−0.281	0.692	0.755	0.188–3.038
Smoking	0.486	0.083	1.626	0.938–2.818	0.276	0.319	1.317	0.766–2.267
Drinking	−0.191	0.548	0.826	0.442–1.543	−0.140	0.651	0.869	0.474–1.595
HTGW	0.509	0.036	1.664	1.033–2.680	0.637	0.007	1.891	1.192–2.998

Abbreviations: *WTI* Waist circumference index,*BMI* Body Mass Index,*95% CI* confidence interval,*OR* odds ratio,*CHD* Coronary heart disease

The left table represents the prediction of organ failure and the right represents the analysis of SIRS

## Discussion

Clinically, there are many possible causes of pancreatitis including biliary, alcohol, and hypertriglyceridemia, of which biliary factors are the most common [31–33]. In China, HTG-AP has replaced alcoholic AP as the second most common cause [23, 27]. Our results showed that 28.2% of patients exhibited biliary AP, 6.7% had alcoholic AP, and 40.2% of patients had HTG-AP, suggesting that hypertriglyceridemia is the most common cause of AP. When doctors judge patients as having biliary AP, they may pay more attention to possible biliary obstruction and fail to measure TG. Thus, these patients would have been excluded for having incomplete data in our study. In subgroup analysis, our data demonstrated that biliary factors were the main cause of AP (51.1%) in the NWNT group. In contrast, the main cause of AP in the HTGW group was hypertriglyceridemia, which accounted for 67% of cases and met the definition of HTGW.

Obesity refers to an excessive deposition of adipose tissue. A meta-analysis of observational results showed that obesity (BMI > 30 kg/m^2^) was a risk factor for SAP, local complications, SIRS, and increased rate of death [34]. Obesity was classified as abdominal or subcutaneous. Current clinical studies show that visceral fat, which exists in or around the pancreas, may worsen the prognosis of AP, whereas subcutaneous fat rarely affects prognosis [35]. Visceral fat is increasingly recognized as a risk factor for AP. Our data clearly showed that patients with SAP comprised 10.8% and patients with SIRS comprised 51.8% of the HTGW group, which was more severe than the composition of the other two groups. This finding is in agreement with earlier results [36, 37], suggesting that WC and hypertriglyceridemia have a clear correlation with AP severity.

It is difficult to measure visceral obesity as the measurements need to be verified using a specific software system [38]. However, such methods are more common in research, whereas their clinical use is limited. Lemieux first introduced the HTGW phenotype in the Quebec Cardiovascular Study and suggested that this phenotype may be an inexpensive tool for identifying asymptomatic patients at elevated risk of coronary heart disease [14]. Use of HTGW is based on the concept that abdominal fat and dyslipidemia are at the heart of the Mets pathogenesis. WC is a simple anthropometric value with good reproducibility, and the measurement of WC is convenient and inexpensive to obtain. However, WC does not distinguish between visceral and subcutaneous fat. Hypertriglyceridemia is a simple clinical marker of excessive visceral fat associated with increased WC. The HTGW phenotype, which indicates people with high WC and hypertriglyceridemia [20], is a simple and reliable phenotypic indicator of metabolic disease related to visceral obesity. This phenotype has been used as a screening indicator for cardiovascular disease, prodromal diabetes, and other diseases. So far, the correlation between the phenotype and AP has not been reported.

Our results indicated that patients with AP who exhibited the HTGW phenotype had a higher incidence of SAP, SIRS, and organ failure. In addition, the phenotype was useful for predicting the occurrence of organ failure and SIRS. Multivariate logistic regression analysis showed that patients with the HTGW phenotype had 1.664 times the risk of organ failure and 1.891 times the risk of SIRS, compared with the other groups. Accordingly, the HTGW phenotype acts as a simple screening tool for early prediction of SAP.

WTI, first described by R F Yang et al., is a quantitative indicator of the HTGW phenotype [15]. Our data showed that, as AP increased in severity, both the proportion of the HTGW phenotype and WTI significantly increased. It is worth noting that BMI, which only represents overall obesity, did not significantly differ among the MAP, MSAP, and SAP groups. This finding confirmed those of Sadr-Azodi; that is, abdominal obesity (rather than total obesity) was an independent risk factor for development of AP [36].

The HTGW phenotype may be closely related to AP severity via free fatty acids (FFAs). Both the lipolysis of adipose tissue and hydrolysis of triglyceride produce excessive FFAs after AP onset. Clinical research revealed that circulating FFAs are positively correlated with the severity of AP [39]. Furthermore, Substantial studies have shown that FFAs, especially unsaturated fatty acids (UFAs), exert damage on pancreatic acinar cells [40–42]. Patel et al. found that visceral fat triglyceride secreted excessive UFAs under the action of pancrelipase. This promoted the development of MAP into SAP, eventually triggered MODF and increased the mortality [41, 43]. Navina S et al. also showed that UFAs, produced by necrotic peripancreatic visceral fat, aggravated pancreatic inflammation and promoted the transformation of MAP to SAP [42]. In addition, orlistat, a esterase inhibitor, could significantly attenuated the AP severity in obesity mice [41–43]. All the previous findings confirm the critical role of FFAs in the pathomechanism of AP patients with obesity.

There were several limitations to this study. Firstly, this was a retrospective analysis, and not all patients had their WC measured by soft ruler at admission. For these patients, WC could be only measured by abdominal CT. Abdominal CT is not an infallible measure of WC, which could have introduced some data bias. However, it still can get relatively accurate data, as described in Additional file 1 Figure S1. Obviously, we cannot directly compare WC obtained by CT with that obtained by soft ruler. Accordingly, the WC data of patients in this study were all obtained by abdominal CT in order to minimize error and avoid bias. Secondly, this was a single-center retrospective study with a small sample size. Multi-center studies are needed to validate our findings and further explore the clinical significance of the HTGW phenotype as an early indicator of SAP in patients with AP.

In summary, our results demonstrate that patients with AP, with the HTGW phenotype, have a higher risk of organ failure. WTI, as a quantitative indicator of the HTGW phenotype, was positively correlated with the severity of AP. The HTGW phenotype and WTI can be used as simple indicators for early identification of high-risk patients with SAP.

## Additional file

Additional file 1: **Figure S1.** After admission, the AP patient underwent CT scan to assess the disease. The waist circumference was regarded as an ellipse in the mathematical model. From the above diagram we can see that the long axis was 311.5 mm and the short one was 208.7 mm on the L4 plane, and the coefficient was × (short axis + long axis) ÷2 according to the ellipse formula. It is concluded that the Waist circumference of the patient is 82 cm. (DOCX 1308 kb)
