# Supplementary material for: Association of the hypertriglyceridemic waist phenotype and severity of acute pancreatitis
Source: Lipids Health Dis. 2019 Apr 9;18:93. doi: 10.1186/s12944-019-1019-2 (PMC6454768; doi:10.1186/s12944-019-1019-2)
Supplement: Supplementary file 1 — Figure S1. After admission, the AP patient underwent CT scan to assess the disease. The waist circumference was regarded as an ellipse in the mathematical model. From the above diagram we can see that the long axis was 311.5 mm and the short one was 208.7 mm on the L4 plane, and the coefficient was × (short axis + long axis) ÷2 according to the ellipse formula. It is concluded that the Waist circumference of the patient is 82 cm. (DOCX 1308 kb) [file 12944_2019_1019_MOESM1_ESM.docx]

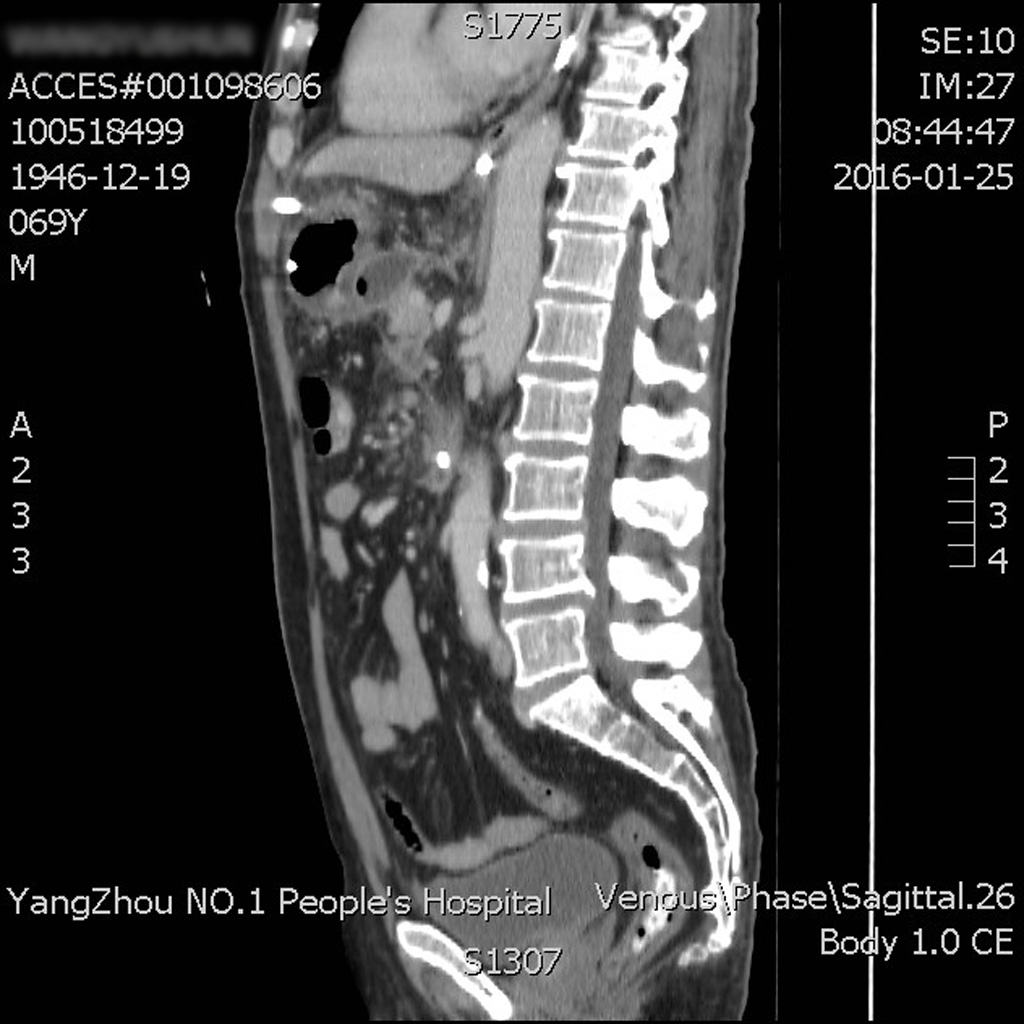

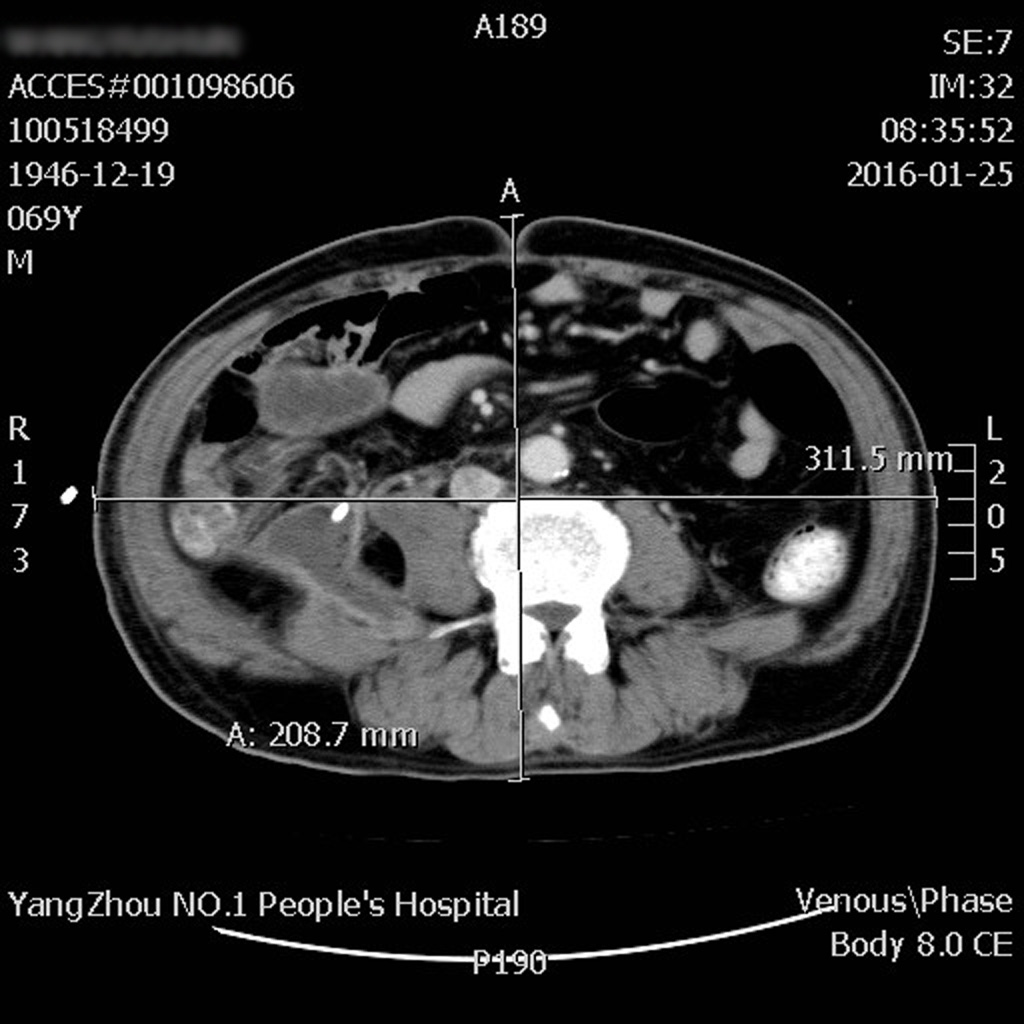


**Supplementary figure 1** After admission, the AP patient underwent CT scan to assess the disease. The waist circumference was regarded as an ellipse in the mathematical model. From the above diagram we can see that the long axis was 311.5 mm and the short one was 208.7 mm on the L4 plane, and the coefficient was × (short axis + long axis) ÷2 according to the ellipse formula. It is concluded that the Waist circumference of the patient is 82 cm.
